# How to Behave at a Party

**Published:** 1881-08

**Authors:** 


### HOW TO BEHAVE AT A PARTY.

I REMEMBER that when I was quite young, going to a party was as much a trial to me as a pleasure. Being diffident, I dreaded entering the room and encountering the eyes of the people already assembled there ; and once fairly in, I was overshadowed all the evening by the dreadful necessity of by-and-by retiring. Besides, I felt a sense of responsibility which was very oppressive, and was so afraid of not doing or saying what was expected of me that I moved and acted awkwardly, and no doubt felt perfectly miserable.

Perhaps some of you may have had experiences similar to mine. Now let me tell you that I have to laugh at my foolish shyness, and to be very sorry for boys and girls who suffer from the same thing. When you are invited to a company, the first thing in order is to reply to the invitation. This is polite, whether you accept or decline, and it is imperative if you decline. Send your answer as soon as possible, in such simple phrase as this: "Harold," or "Florence, thanks Mrs. —— for her kind invitation for Thursday evening, and accepts it with pleasure," or "declines it with real regret," as the case may be. Arrived at your friend's house, you will be directed to the proper place for the removal of your wraps and the arrangement of your toilet, and then you have only to proceed to the parlor, where your hostess will relieve you from embarrassment by meeting you at once. She is, of course, the first person whom you are to greet. Having spoken to her, you are at liberty to find other friends.

Do not think that people are looking at you, or noticing your dress or your looks. They are doing nothing of the kind. Engage heartily in whatever amusement is provided for the occasion, but do not put yourself needlessly forward. If spoken to, reply modestly but intelligently, even though for a moment there be a hush in the room. If you really wish to enjoy yourself, seek out some one who seems more a stranger than yourself, and try to do something for his or her pleasure. Forget you are not acquainted with everybody, and remember it is your duty to help your hostess in making her party a success. Should your greatest enemy be present, you must, of course, be perfectly civil and agreeable in your manner to him, for in your friend's house you are under a flag of truce.

When you say good-night to your entertainers, be sure to thank them for the pleasure you have had. Do not stay too late, but avoid being the first to go ; or if you must leave early, do it as quietly as possible, lest your withdrawal should be the signal for others to leave, thus breaking up the party too soon.

